# Using collision cones to assess biological deconfliction methods

**DOI:** 10.1098/rsif.2016.0502

**Published:** 2016-09

**Authors:** Natalie L. Brace, Tyson L. Hedrick, Diane H. Theriault, Nathan W. Fuller, Zheng Wu, Margrit Betke, Julia K. Parrish, Daniel Grünbaum, Kristi A. Morgansen

**Affiliations:** 1 William E. Boeing Department of Aeronautics and Astronautics, University of Washington, Seattle, WA, USA; 2 School of Aquatic and Fishery Sciences, University of Washington, Seattle, WA, USA; 3 School of Oceanography, University of Washington, Seattle, WA, USA; 4 Department of Biology, University of North Carolina, Chapel Hill, NC, USA; 5 Department of Computer Science, Boston University, Boston, MA, USA

**Keywords:** collision avoidance algorithm, animal behaviour, velocity obstacles, collision cones, nonlinear control, multi-species comparison

## Abstract

Biological systems consistently outperform autonomous systems governed by engineered algorithms in their ability to reactively avoid collisions. To better understand this discrepancy, a collision avoidance algorithm was applied to frames of digitized video trajectory data from bats, swallows and fish (*Myotis velifer*, *Petrochelidon pyrrhonota* and *Danio aequipinnatus*). Information available from visual cues, specifically relative position and velocity, was provided to the algorithm which used this information to define collision cones that allowed the algorithm to find a safe velocity requiring minimal deviation from the original velocity. The subset of obstacles provided to the algorithm was determined by the animal's sensing range in terms of metric and topological distance. The algorithmic calculated velocities showed good agreement with observed biological velocities, indicating that the algorithm was an informative basis for comparison with the three species and could potentially be improved for engineered applications with further study.

## 1. Introduction

An increasingly wide array of unmanned vehicles is becoming available with a range of form factors and ever-expanding capabilities. Fully using the enhanced control authority and sensing that comes with these improvements in order to effectively and safely navigate the world requires improved control algorithms and estimation strategies capable of real-time path planning through changing conditions and unexpected obstacles. Many biological systems display a remarkable ability to perform such tasks during natural behaviour [1], so we look to their example to gain insight into the principles underlying the highly effective reactive collision avoidance abilities developed through evolution.

The study of systems theory with respect to biological behaviour, and vice versa, has been a popular topic for many years that has resulted in a number of advances in the understanding of biological systems and capabilities for engineered systems. Biological models have been developed to describe the emergence of self-organization and other collective behaviour in large groups of animals, including schooling and shoaling fish, flocking birds, swarming insects and herding land animals [2]. A particle model was used to produce realistic flocking animations by basing the behaviour of boids (bird-oid objects) upon the requirements of collision avoidance, velocity matching and flock cohesion [3]. Consensus decision-making of social groups has been studied to investigate decision-making among a group [4,5]. Not limited to studies of collective behaviour, Karaman & Frazzoli [6] developed a theoretical framework to investigate the collision avoidance problem solved by birds when navigating a forest at high speed and found a critical speed below which a conflict-free path could be flown indefinitely.

Certain models provide insight into animals' sensing or cognitive limitations, which clearly impact collision avoidance capabilities. For example, animals in a large group appear to only take into account some smaller number of nearby animals. The membership of, and interactions within, these smaller groups may be dependent on a metric distance, for example, the zonal model of shoaling fish [7], or a topological distance, as in the model of flocking starlings [8]. Models of perception have also been developed to investigate the use of optic flow in birds [9] and bats [10] and even visual tracking to aid collision avoidance in insects [11].

Typically, these models begin with extensive observations of the species and develop into theories that explain or reproduce the behaviour—the study here takes the opposite approach, by starting with an engineered algorithm and determining how well it correlates to observed behaviour. Dynamic collision avoidance for autonomous vehicles is challenging and is an ongoing topic of research. Preventing vehicles from coming too close to static objects in a cluttered environment can be done, however, it becomes much more challenging when dynamic obstacles are introduced since offline or global path planning techniques can no longer be used. A wide array of methods have been applied to the problem of conflict resolution, i.e. the act of identifying and avoiding future collisions, with varying degrees of centralization and safety and convergence guarantees [12]. Centralization refers to the distribution (or lack thereof) of information and processing; a distributed system falls between the extremes of global and local information and processing, with each agent processing global information individually. Prescribed manoeuvres, e.g. always turn left, and force field methods, in which goals and obstacles are treated as oppositely and similarly charged particles, have been shown to work, but can become prohibitively complex for a large number of vehicles [13,14]. Optimization methods seek to find the best collision-free path with respect to some cost function. The optimal solution can be found for all cooperative agents using a centralized approach [15] or for individual agents using a decentralized [16] or distributed [17,18] approach.

Parallels in biology can be drawn to some conflict resolution algorithms, including model predictive control, the so-called bug-family algorithms and certain sensor-based techniques. A model predictive control strategy plans a safe path based on a goal for a limited time horizon, executes the first step of that plan, collects new information, checks for differences between planned and actual outcomes, and repeats the process [19]—not unlike how biological species process information [20]. Bug algorithms use a strategy requiring minimal sensing information: head towards the target until an obstacle is detected, then follow its boundary until the path to the target is clear again [21]. Sensor-based techniques rely on knowledge of some region of space near the autonomous agent that can be sensed and used for planning; *velocity obstacles* fall into this category, using relative position and speed of other agents and obstacles to discriminate safe and unsafe velocity vectors [22,23].

The Distributed Reactive Collision Avoidance (DRCA) algorithm uses collision cones, a version of velocity obstacles, to provide safe paths for an arbitrary number of agents [18]. The algorithm detects conflicts (future collisions) by checking if the current velocity falls within a collision cone. If a conflict is detected, a safe velocity nearest the original velocity is found and implemented in a deconfliction manoeuvre. In biological terms, this implementation assumes an animal will continue in one direction until something in its environment forces it to change, and then it will deviate as little as possible from its original path. The calculations are performed for each agent individually and require only information that can be obtained from visual cues: velocity, bearing and range of other agents. Visual input is noted here as it is common to all species under study; however, for the purposes of the following analysis the relative velocity and/or position information could come from any sensory input. The distributed nature, lack of complicated cost function and limited sensing requirements of this algorithm make it a good candidate for comparison with biological species. Furthermore, both birds and bats are known to use velocity cues in target tracking by applying a version of constant bearing, decreasing range techniques [24,25].

In this work, we compare the collision avoidance trajectories of bats (*Myotis velifer*), birds (*Petrochelidon pyrrhonota*) and fish (*Danio aequipinnatus*) to those predicted by the DRCA algorithm. This paper presents an extension of the original DRCA algorithm from two to three dimensions and corrects a previous study done by Boardman *et al.* [26], which performed a similar analysis by flattening the trajectories to two dimensions and applying the original DRCA algorithm. The three-dimensional analysis provides the algorithm with spatial information that corresponds more closely to the three-dimensional environments of the animals and eliminates ‘phantom conflicts’ created by two-dimensional conflict checking. We hypothesize that all these species will approximately match the DRCA predictions due to the overlap between DRCA processing and sensory requirements and those likely available to the animals. We further hypothesize that the birds and bats will agree more closely with the DRCA predictions than the fish because one of the DRCA assumptions—the desirability of a future trajectory close to the current trajectory—is more likely satisfied for fast moving flying animals in a diffuse fluid than for slower animals in a dense fluid.

## 2. Material and methods

### 2.1. Biological species

The species used for comparison in this study were chosen due to their agile manoeuvring capabilities and social nature.

#### 2.1.1. Emerging cave bats

*Myotis velifer* are bats that roost in colonies found in caves and tunnels. Each night they emerge in small groups after sunset [27]. They are insectivores and use echolocation as their primary means of sensing, although vision may also play a role in navigation [10]. An average bat in this species is 0.1 m long with a wingspan of 0.3 m, weighs 12 g, and has a cruising speed of 5.5–6.6 m s^−1^ [27,28].

**Figure 1.**
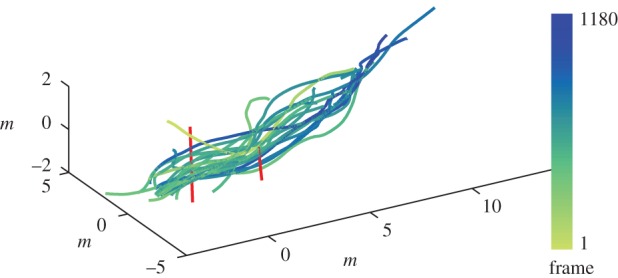
Trajectories of 20 bats emerging from their cave (blue-green lines; data point colour indicates frame number) and static objects within the frame (red lines). Trajectories of the same colour were present concurrently. Data are shown before smoothing was applied.

Trajectory data were collected for 9 s and 79 s at 131.5 frames per second as groups of 20 and 86 bats, respectively, emerged from their cave on the Bamberger Ranch in Texas. Each bat was in view for approximately 1.7 s with a maximum of 10 bats in view at any one time. In addition to other animals, there were also two static objects in their flight path: a pole 1.6 m tall and a vine 0.2 m wide extending from the ground to the forest canopy. The bats' trajectories and the static objects are depicted in figure 1.

The cave bats were filmed in low light using three mid-infrared FLIR SC8000 cameras at a resolution of 1024 × 1024 pixels [26]. Camera calibration was performed for both bats and swallows (see below) by passing a 1 m long reference object through the shared field of view and applying a structure-from-motion algorithm [29]. Correspondences between the views were made using semi-automated tracking tools, and the three-dimensional trajectories were generated using least-squares reconstruction. The bats were filmed by the Kunz Bat Lab, and the pre-experiment planning of the multi-camera recording and the post-processing of the three video streams was done by the Image and Video Computing Group, both at Boston University.

#### 2.1.2. Manoeuvring swallows

Cliff swallows (*Petrochelidon pyrrhonota*) are colonial passerine birds that tend to nest on cliffs and beneath overhangs. They are capable fliers, interacting with the elements of their environment as well as with each other and foraging for insects. Cliff swallows are typically 0.13 m long with a wingspan of 0.29 m, weigh 24 g and have an average cruising speed of 5.3 m s^−1^ [30].

**Figure 2.**
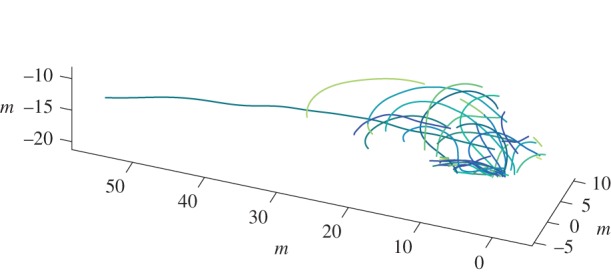
Trajectories of 43 cliff swallows from one bridge site recording. Each colour represents an individual animal's trajectory.

Swallow trajectory data were collected near a nesting site at the North Carolina Highway 751 bridge over Jordan Lake, Chatham County, NC, USA. Each scene was recorded for 4.75 s at 100 frames per second. The scenes examined here include 21–51 individual swallow trajectories. Trajectories of the cliff swallows are shown in figure 2.

The swallows were filmed using three IDT N5r cameras outfitted with Nikon 20 mm f/28 AF lenses at a resolution of 2336 × 1728 pixels [31]. Calibration was performed using the same method as for the bats. Objects were tracked and triangulated between two and three of the cameras to produce the three-dimensional data [32]. The swallow data were collected by the Hedrick Lab at the University of North Carolina at Chapel Hill.

#### 2.1.3. Shoaling fish

*Danio aequipinnatus* are small freshwater fish found in loose groups in rivers and streams. The animals in the trial were around 0.053 m in length with a circumference of approximately 0.15 body lengths (8 mm) and weighed 1.7 g [4].

**Figure 3.**
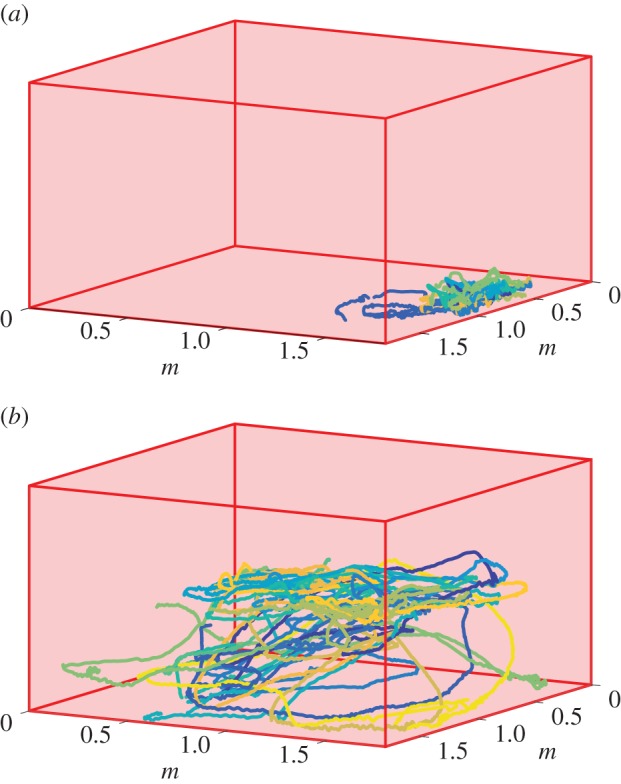
Trajectories of giant danio (blue-green lines) depicted within their fish tank (red surfaces); shown are (*a*) untrained giant danio prior to the food stimulus and (*b*) trained giant danio after the food stimulus. Each colour and line represents an individual animal's trajectory; all animals were present throughout the recording.

The fish were filmed in a square tank measuring 1.8 m per side and 1 m deep. During each recording, there were 15 fish in the tank, and the schools were some combination of naive fish and fish trained to respond to a food stimulus. Specifically, during the two weeks prior to the data collection, the trained fish were exposed to red lights when food was delivered to the tank. The recordings were captured at 30 frames per second and separated into 1 min segments before, during and after the food cue [4]. To minimize the variables for the current study, only datasets with all untrained or trained fish before and after the food cue were used for analysis; trajectories are shown in figure 3*a*,*b*.

The movements of the fish within the tank were captured by four Panasonic x300 digital palmcorder cameras [4]. The video data were processed into three-dimensional trajectories using the software package tracker3D. Camera calibration was done using a grid placed on the top and bottom of the tank. The fish data were collected in the Birdfish Lab under the direction of Julia Parrish at the University of Washington.

#### 2.1.4. Smoothing

All position data were smoothed using a quintic spline and Butterworth filter. The fourth-order Butterworth filter was used to identify higher frequency content associated with noise and wingbeats, with cut-off frequencies of 8 Hz for bats and birds and 6 Hz for fish. This high-frequency content was then used to set the tolerance for a smoothing quintic spline. After smoothing, the velocities were calculated using finite differencing.

### 2.2. Behavioural models

The animals may not pay attention to all of the objects around them so two models, based on metric and on topological distance, are presented for selecting an influential subset of objects in each scene to use for the collision avoidance calculations.

#### 2.2.1. Metric distance

**Figure 4.**
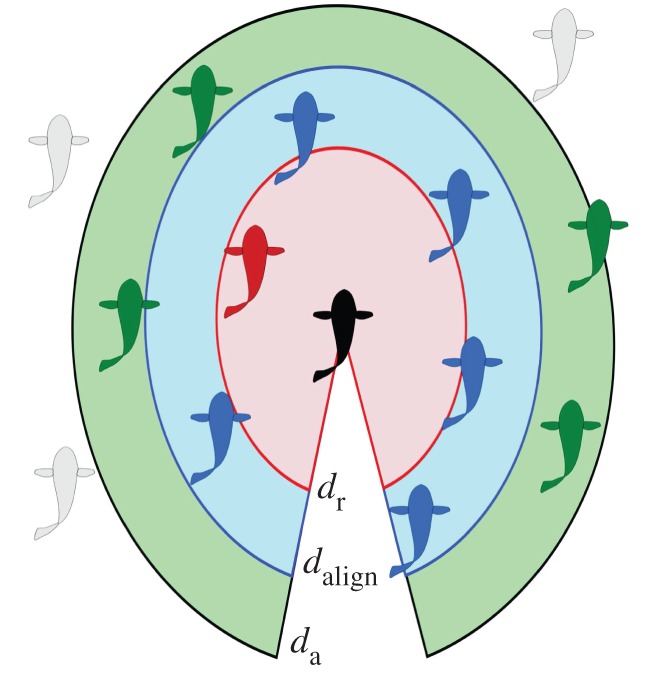
In the zonal model, radii around the focus animal (black) define the type of interaction with other animals: those within *d*_r_ (red) are repulsive, those between *d*_r_ and 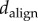 (blue) are neutral or aligning, and those between 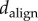 and *d*_a_ (green) are attractive. Animals outside of *d*_a_ (grey) are not sensed or are ignored.

Also called a zonal model, this method uses the radial distance from an animal to classify its interaction with nearby neighbours as repulsive, aligning or attractive [7], as depicted in figure 4. The outermost zone within the sensing range of the animal is the zone of attraction. The simplest variation of this model, which uses spherical zones, was used for the following analysis. The radius of the sphere, the (constant) distance of attraction, *d*_a_, was used to define the maximum sensing range within which potential collisions are, presumably, detected and avoided. For this study, *d*_a_ = 3 m for the flying species and *d*_a_ = 3 m for the fish.

#### 2.2.2. Topological distance

**Figure 5.**
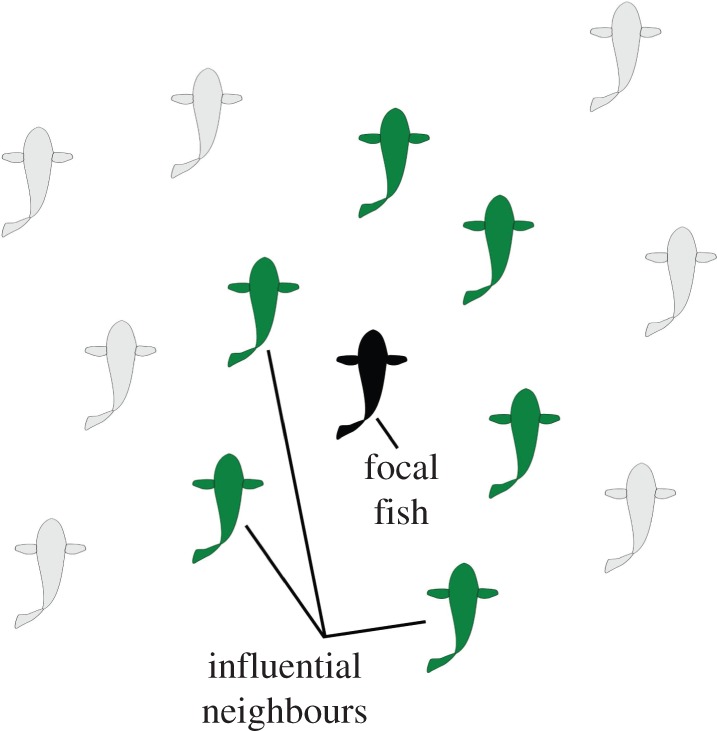
In the topological distance model, a set of animals nearest the focal animal (black) is considered to comprise influential neighbours (green). Animals outside this inner grouping (grey) are not considered by the focal animal.

The topological distance is described by relative proximity of animals to the focus animal—this distance is measured in bats/birds/fish rather than metres, as depicted in figure 5. The number of animals under consideration remains constant regardless of the radial distance from the focus to nearby animals. Thus, the cognitive load remains relatively constant for any density of the group, and the animal does not become overwhelmed if a contraction of the group occurs, for instance. The number of influential neighbours has been shown to be important in both flocking birds [8] and schooling fish [7]. In birds, models best match observed data when six to seven influential neighbours are considered, which is consistent with the ability of pigeons to track objects in sets of fewer than seven [33]. For a model of schooling fish, Viscido *et al.* [7] showed a relationship between the number of influential neighbours and group size and that group size is in turn related to population size; based on the Viscido model, the number of influential neighbours for a population of 15 is seven. The subsequent analysis considers a range of maximum nearest neighbours, from the single closest animal up to all animals within sensing range.

### 2.3. Collision avoidance

Avoiding obstacles is a common goal of mobile systems, both biological and engineered. The DRCA algorithm is based on two layers: a deconfliction manoeuvre that brings vehicles out of conflict by choosing the safe velocity nearest to the current velocity and a deconfliction maintenance manoeuvre that ensures conflicts do not occur in the future [18]; here, only the former component is used. The algorithm begins by checking to see if a conflict exists. If a conflict exists, a deconfliction manoeuvre uses the current velocity and position information of the focus agent and all other agents within sensing distance to find a safe velocity that requires minimum deviation from the original velocity.

#### 2.3.1. Conflict definition and detection

Impending collisions must first be perceived before they can be avoided, so the first step in avoiding collisions is detecting conflict. A *collision* occurs when two objects, *i* and *j*, come within a specified minimum separation distance, 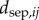. The minimum separation distance, 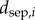, describes the radius of a sphere around object *i*; the minimum separation distance for two objects *i* and *j* is then 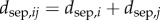. Thus, a collision between objects *i* and *j* separated by the relative position vector 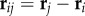 happens when 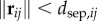.

**Figure 6.**
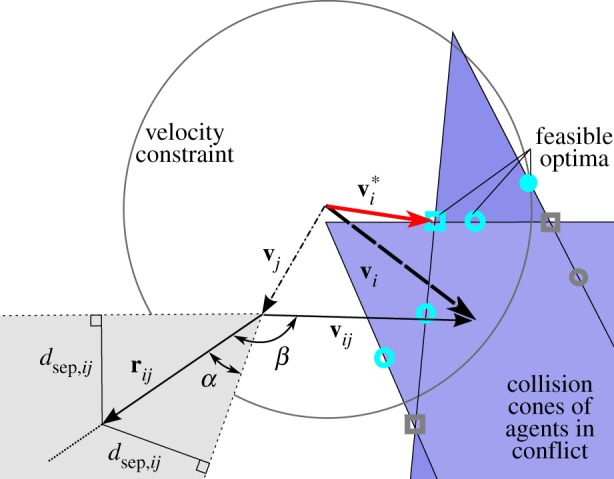
The grey cone with dashed borders depicts the anatomy of a collision cone for an agent that is not in a conflict: 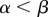 indicates that 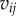 falls outside the collision cone. The blue cones with solid borders depict collision cones for agents in conflict; potential optima are shown that are within the maximum velocity constraint (cyan) and as well as those that are infeasible (grey) at locations 1 and 2 on a single collision cone (open and closed circles, respectively) and location 3 on the intersection of collision cones (square).

**Figure 7.**
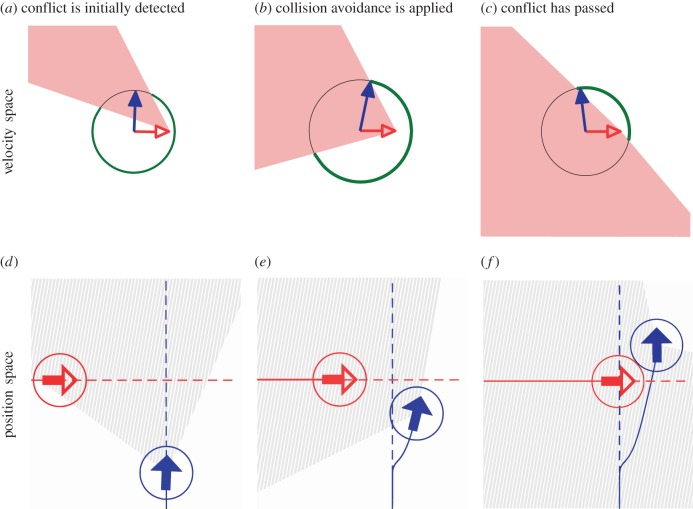
The sequence of figures (progressing from left to right) shows the evolution of an encounter with one agent (blue, solid arrows) using the DRCA algorithm to avoid conflict with another agent (red, open arrows). Figures (*a*–*c*) are in velocity space showing the collision cones for the scenarios depicted in position space in figures (*d*–*f*). The circles shown in velocity space correspond to the momentary speed of the agent following DRCA; the green highlighted arcs indicate safe velocities while the regions within the collision cones correspond to unsafe velocities, denoted in position space by the hashed region.

A *conflict* occurs when two objects will eventually enter a collision if their current velocities are maintained. The set of velocities that will eventually cause a vehicle to collide with another can be described by a collision cone. The collision cone is constructed in velocity space with its vertex at the velocity of the obstacle, 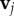, and axis in the direction of the relative position vector, 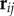; objects *i* and *j* are in conflict if 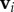 falls within the collision cone generated by 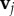. The half-angle of the collision cone, *α*, is calculated from the minimum separation distance and the current distance between two objects by
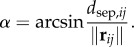
Defining *β* as the angle between the relative position and relative velocity vectors, that is 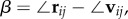 where 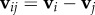, the criterion for conflict is simply 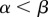 [18]. The collision cone geometry for objects in and out of conflict is shown figure 6, and stages of conflict identification and resolution are shown in figure 7.

Note that the collision cone geometry also provides a method of intercepting another agent: selecting the velocity at the intersection of the collision cone axis with the circle of constant speed is equivalent to a constant bearing, decreasing range strategy.

#### 2.3.2. Deconfliction algorithm

When a conflict is detected, the optimal safe and feasible velocity is found. This velocity is selected by the algorithm to be outside of all collision cones (making it safe) and to require the smallest deviation from the current velocity (making it optimal) while satisfying certain speed constraints dependent on the manoeuvre type (making it feasible). The variable speed manoeuvre requires that the velocity remain below some maximum speed, allowing for changes in both speed and direction. Once the potential optima, 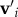, are found, they are put in order from smallest to largest deviation from the original velocity, 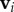, and then each 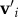 is checked for conflicts. By construction of the ordering, the first conflict-free 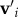 is optimal. The potential optima are found at certain locations defined by the geometry of the collision cone(s) and velocity constraint, represented by the sphere of maximum velocity including the nearest point to 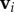, on

(1) a single collision cone,
(2) the intersection of a collision cone and the spherical shell of maximum speed,
(3) two collision cones or
(4) the intersection of two collision cones and the sphere of maximum speed.

In figure 6, locations 1–3 are depicted in a two-dimensional version of the algorithm. Methods for finding all optima, which use a combination of closed-form solutions and optimization equations for numerical methods, can be found in [34].

The evolution of a variable speed deconfliction manoeuvre for a single agent using the DRCA algorithm is shown in figure 7. The conflict begins with the red agent travelling horizontally moving into the detection range of the blue agent which is travelling vertically. The velocity of the blue agent lies within the collision cone generated by the red agent, indicating a conflict (figure 7*a*,*d*). To avoid collision, the blue agent chooses a new velocity at the edge of the collision cone nearest the original velocity; in this case, the blue agent both adjusts its heading clockwise and increases its speed (figure 7*b*,*e*). Once the blue agent has passed the red agent, potential conflicts are behind it, and it can return to its original trajectory (figure 7*c*,*f*).

### 2.4. Data analysis

#### 2.4.1. DRCA with trajectory data

To generate the basis for comparison with the trajectory data, each animal was in turn considered as the focus (agent *i*) with the other animals within the sensing range considered as dynamic obstacles. For each frame in which the focus animal was present, conflicts were checked for the positions and velocities of all obstacles within sensing range. The sensing distance was determined by a combination of metric and topological distances, so only the nearest *N* animals within *d*_a_ of the focus were considered. If a conflict was present, the deconfliction algorithm was used to calculate the optimal velocity, 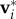. If animals were already closer than the minimum separation distance and thus considered in a collision, the frame was excluded from consideration. Also, to exclude any swallows attempting to land, the DRCA was not applied to frames where swallow velocity was less than 3 m s^−1^.

The algorithmic parameters 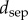 and 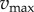 were determined based on the biological characteristics of each species. The minimum required separation distance for each of the flying species was approximately half of the wingspan, 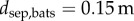 and 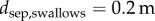, and for the fish it was half of a body length, 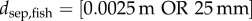. The speed for the maximum velocity requirement was set higher than the typical cruising speed found in the literature for the bats and swallows, at 11 and 18 m s^−1^, and based on the maximum used in models of fish behaviour set at 0.6 m s^−1^ (12 body lengths per second [7]).

The analyses were performed with and without maximum acceleration bounds on the DRCA velocity. Acceleration bounds were applied by scaling the difference between the original velocity and DRCA optimal velocity, that is 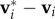, to a maximum acceptable change between frames and using the scaled value as the optimal for comparison. This scaling was done to provide more interpretable results in cases where DRCA called for a dramatic acceleration because accelerations in the algorithm are not constrained and have no optimization cost, whereas accelerations by animals are both constrained and costly. Maximum accelerations for each species were set based on the observed frame-to-frame accelerations. For the flying species, this value was equivalent to maximum accelerations of three times gravity, or 0.23 and 0.3 m s^−1^ change in speed between frames for the bats and swallows, respectively, and 0.05 m s^−1^ change between frames for the fish.

#### 2.4.2. Comparison metric

**Figure 8.**
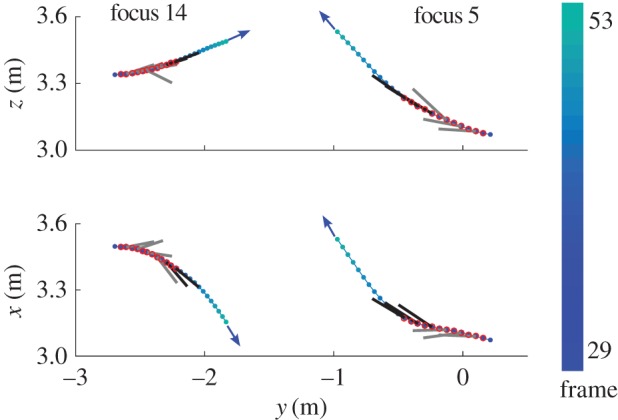
Side and overhead view of two swallow trajectories (blue markers indicate positions, with colour indicating frame number) including a series of conflicts (red circled points). The DRCA optimal velocities without acceleration bounds are shown as exaggerated lines for correlated (black) and uncorrelated (grey) frames.

For every conflict, each DRCA optimal velocity, 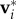, was compared to the observed velocity, 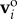, defined as the velocity required to get to the focus animals' position 50 ms later; this time lapse was chosen to approximate the animal's reaction times, as around half of a locomotor cycle for the flying species [35,36] and one-sixth of a tail beat cycle for the fish [37]. The metric used was the normed difference between the velocity vectors in three dimensions, 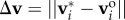, and was calculated for each frame in which the focus animal experienced a conflict. The correlation percentage was defined for each animal as the portion of 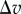 falling below a threshold, 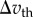, defined as 1 m s^−1^, or half of 1 s.d. of velocities, for the flying species, and 0.4 m s^−1^, or 1 s.d., for the fish. The mean and standard deviation of the correlation percentage for all animals that experienced at least one conflict was calculated for each species. Interactions between animals typically lasted multiple frames, and each animal involved had a unique 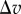; an example encounter between two swallows is shown in figure 8.

## 3. Results

**Figure 9.**
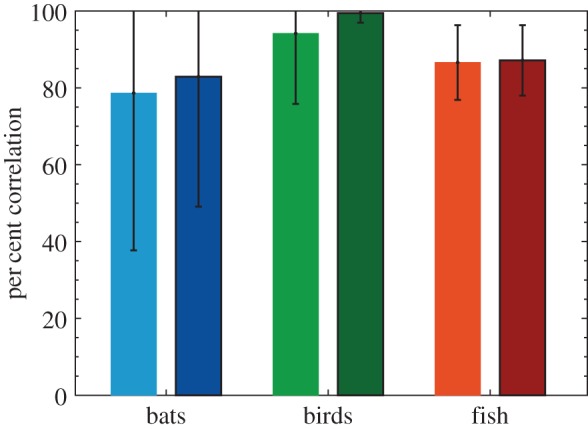
The per cent correlation for each species with (darker bar with border on the right) and without (lighter bar on the left) acceleration limits applied to the DRCA optimal velocity. The error bars indicate one standard deviation above and below the mean per cent correlation (error values above 100% are not shown).

In general, avoidance manoeuvres in all three species agreed with the DRCA optimal velocity. Following our basic standard for agreement (see §2.4.2), the swallows were in agreement more than 90% of the time, fish greater than 80% of the time and bats greater than 70% of time (figure 9).

Agreement between DRCA and observed animal motion was enhanced in all cases by limiting the maximum acceleration used in the avoidance manoeuvre (figure 9), although in most cases the improvement in agreement between model and animal was slight.

**Figure 10.**
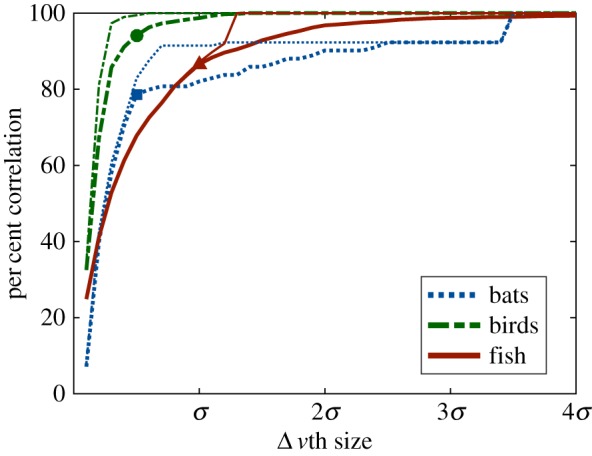
The per cent correlation as a function of 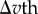 for bats (blue dashed), birds (green dashed-dot) and fish (red solid) with (thin line) and without (thick line) acceleration limits applied to the DRCA optimal velocity; markers indicate values that appear in figures 9 and 11. The *x*-axis is scaled by the standard deviation for each species, which is 2 m s^−1^ for the flying species and 0.04 m s^−1^ for the fish.

As expected, increasing the stringency of the standard for agreement between DRCA and animal motion reduced the overall correlation between the two (figure 10). The swallows showed the highest correlation at all levels; however, the relative order of the bats and fish changed around a single standard deviation.

**Figure 11.**
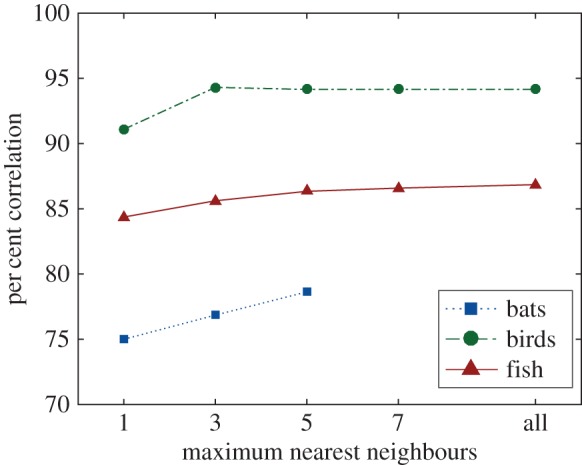
The per cent correlation for bats (blue squares with dashed line), birds (green circles with dashed-dot line) and fish (red triangles with solid line) without acceleration limits applied to the DRCA optimal velocity for each species for a range of maximum topological distances ranging from one to all possible animals within the metric range (3 m for flying species and 0.3 m for fish).

Increasing the topological distance from considering a single animal to all nearby animals does lead to an increase in correlation between DRCA and the biological data for up to seven nearest neighbours in all species (figure 11). The size of the improvement becomes small after considering five nearest neighbours; however, there was a dearth of instances of more than three nearest neighbours in the swallow data and a lack of data points for more than five nearest neighbours in the bat data when limited to the 3 m metric range.

## 4. Discussion

Our initial hypotheses that animal motion would agree with DRCA optimal velocity was supported—we found marked correlation between DRCA optimal velocity and animal motion in three evolutionarily, physiologically and biomechanically distinct vertebrate species.

Our further hypothesis that DRCA-like avoidance manoeuvres would be most common in flying species (birds and bats) versus swimming species (fish) was not supported. The birds (Cliff swallows) did have the highest overall agreement with DRCA, but bats (cave bats) had the lowest. The factors that produced the discrepancy are not clear, but it may relate to the different sensory systems employed by birds versus bats (vision versus vision and sonar) or the different behavioural contexts represented in the data. The swallows were in a mixed flock with birds moving in all directions, whereas the bats were in a directional flock with all animals generally moving in the same direction; this overall flock direction may add an additional constraint to behaviour that reduces correlation with DRCA. Despite the algorithm preventing group alignment or attraction behaviours to meet its primary objective of avoiding collisions, the correlations are still quite good.

Topological distance has been shown to be more important in animal group behaviour, however, metric distance may be important with regard to collision avoidance, and we examined fit to DRCA over a range of metric distance thresholds (see the electronic supplementary material, figure). In general, results parallel the topological analysis because the conditions largely overlap, e.g. a small metric distance threshold is likely to include only one or two topological neighbours, so increases in the metric threshold correspond to increasing agreement with DRCA in all species. One exception lies in bats, which exhibit maximum agreement at a non-maximal metric threshold, suggesting that metric information may play a role in this species or behaviour; confirmation would require a larger dataset with a greater range of discordant metric and topological thresholds.

Although all three species correlated with DRCA, we do not suggest that the animals are actually using this algorithm. Instead, we believe that because DRCA operates from a single agent perspective using limited sensory information, it naturally matches many of the limitations under which animals operate and therefore produces similar behaviour in many cases. Thus, investigation of specific cases of agreement or disagreement between DRCA optimal velocity and actual animal motion is likely to reveal other trade-offs under which animals operate. For example, flying animals may prefer avoidance manoeuvres that preserve total (kinetic and potential) energy, flying up when slowing and down when speeding up, to avoid having to recreate that energy from metabolic supplies later. The DRCA algorithm could potentially be used to investigate ‘higher stakes’ cases of collision avoidance, such as chases or predator–prey interactions, by comparing the optimal velocity to that of the fleeing animal; specifically, increasing the required 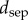 would simulate a greater desired separation or higher risk scenario and generate larger magnitude changes in velocity correspondingly.

From the engineering perspective, a similar, more detailed study of individual conflicts could be used to identify cases where there are smaller or larger deviations between the algorithm and the observed behaviour in order to determine what situations lead to more or less agreement. This investigation could then inform an engineered system when to follow the algorithm and when to employ more or less conservative actions based on biological examples.

## Supplementary Material

Supplementary Figure: Correlation rate as a function of metric distance

## Data accessibility

Data are available from the Dryad Digital Repository at: http://dx.doi.org/10.5061/dryad.533gf [38].
